# Root canal segmentation from cone-beam computed tomography guided by micro-computed tomography based on deep learning

**DOI:** 10.1186/s12903-026-07918-2

**Published:** 2026-03-10

**Authors:** Xianhua Gao, Jingzhi Ma, Bo Li, Yimeng Fang, Lianting Hu, Min Zhou, Bing Fan

**Affiliations:** 1 State Key Laboratory of Oral & Maxillofacial Reconstruction and Regeneration, Key Laboratory of Oral Biomedicine Ministry of Education, Hubei Key Laboratory of Stomatology, School & Hospital of Stomatology, Wuhan University, 237 Luoyu Road, Wuhan, 430079 China; 2 Department of Stomatology, Tongji Hospital, Tongji Medical College, Huazhong University of Science and Technology, Wuhan, China; 3 The Data Center, Wuhan Children’s Hospital (Wuhan Maternal and Child Healthcare Hospital), Tongji Medical College, Huazhong University of Science and Technology, Wuhan city, Hubei Province 430016 China; 4 Department of Physics and Astronomy, University College London, London, United Kingdom; 5 Department of Industrial and Systems Engineering, Wuhan University of Science and Technology, Wuhan City, Hubei Province China

**Keywords:** Deep learning, AI segmentation mode, Root canal segmentation, Cone-beam computed tomography, Micro-computed tomography

## Abstract

**Background:**

Accurate root canal segmentation from cone-beam computed tomography (CBCT) is essential for endodontic diagnosis and treatment planning. This study aims to explore the feasibility of using deep learning (DL) models, trained on CBCT images of extracted teeth guided by micro-computed tomography (µCT), for clinical CBCT image segmentation.

**Methods:**

A dataset of 56 extracted teeth with diverse root canal complexities was constructed, combining CBCT and µCT scans. Ground truth annotations were derived from µCT-based masks and registered to CBCT images. DL models based on U^2^-Net architecture were trained and evaluated for tooth and root canal segmentation, comparing µCT-guided and manual-label-based approaches. We further investigated the impact of voxel size and image resampling on performance. Finally, the trained models were applied to segment root canals in clinical CBCT images, with the results validated by endodontic specialists.

**Results:**

The µCT-guided AI segmentation method outperformed the manual-label-based approach. Utilizing a smaller native voxel size (80 μm), coupled with image resampling, proved particularly advantageous for capturing intricate anatomical details. In clinical validation, the model delivered rapid and accurate root canal segmentation, with 94% of single-rooted teeth and 100% of molars rated as “excellent” or “good”.

**Conclusions:**

Results demonstrated the potential of µCT-guided AI models for enhancing root canal segmentation in clinical practice, offering a promising tool for digital dentistry.

**Clinical trial number:**

Not applicable.

## Background

A thorough understanding of root canal anatomy is fundamental to the success of endodontic treatments [1]. Although micro-computed tomography (µCT) is considered the gold standard for high-resolution ex vivo analysis of root canal systems [2–4] its clinical application is limited due to high radiation dose, long scan time, and a small field of view (FOV).

Cone-beam computed tomography (CBCT) has emerged as a practical alternative to medical CT in dental imaging, offering multi-planar visualization. A critical factor is voxel size, which is inversely related to the field of view (FOV): a smaller FOV allows for higher resolution (smaller voxel size), enabling detailed visualization of fine anatomical structures [5, 6]. Its advantages include shorter scanning times and lower radiation doses, making it suitable for clinical 3D dental radiology [7, 8]. Despite these benefits, CBCT images often suffer from blurring and are presented as two-dimensional sequences, requiring clinicians to rely heavily on their experience to interpret the internal 3D structure of teeth. Consequently, there is a pressing need to advance automated and precise root canal segmentation (RCS) techniques from CBCT images, particularly for applications in digital dentistry such as guided endodontics [9–11].

The segmentation of CBCT images presents several challenges, including noise, limited resolution, beam hardening artifacts, and the inherent morphological variability of dental anatomy [12]. Traditional methods, such as simple thresholding (effective for µCT but not CBCT) [2], level sets (prone to over-segmentation) [13], graph cuts (requiring user interaction) [14, 15], and template-based fitting (struggling with multi-rooted teeth) [16], often lack the required robustness and precision.

Deep Learning (DL), a subset of artificial intelligence (AI), has recently gained significant attention for its potential in medical imaging [17–19]. By leveraging Convolutional Neural Networks (CNNs), DL can integrate both low- and high-level features, outperforming traditional methods in accuracy and speed [20–23]. However, its application to RCS from CBCT remains limited [24, 25]. Previous studies have explored various DL architectures (e.g., U-Net, residual U-Net) for this task [26–30], but accurately capturing intricate apical anatomy remains difficult. Some efforts have focused on specific tooth types or relied on manual annotations as ground truth, which are time-consuming and subject to observer variability [31].

A critical challenge for supervised DL is obtaining high-quality ground truth. An innovative approach involves using automatically generated µCT segmentations (via thresholding) to guide the training of models for CBCT segmentation through image registration [30]. However, the generalizability of such models can be constrained by limited training data diversity, often comprising only specific tooth types (e.g., premolars).

This study aimed to address these gaps by leveraging the Tooth and Root Canal Morphology Database (School of Stomatology, Wuhan University, China), which contains over 3,000 extracted teeth with corresponding µCT scans. Our objectives are twofold: First, to develop and compare DL models for RCS from CBCT images of extracted teeth, using both µCT-guided and manual-label-based training strategies, while evaluating the impact of FOV and interpolation algorithms. Second, to apply the best-performing model, utilizing the established CBCT-µCT mapping, to segment root canals in clinical patient CBCT images. The performance will be validated by endodontic specialists to assess its clinical feasibility.

## Methods

### Samples and data collection of isolated teeth

#### Teeth samples

A total of 56 extracted teeth, comprising 28 single-rooted teeth (SR) and 28 molars (M), were selected from the Tooth and Root Canal Morphology Database. All selected teeth(26 anterior teeth, 2 premolars, 9 maxillary molars, and 19 mandibular molars with C-shaped canal configurations)exhibited fully formed roots and were free from cracks, restorations, root fillings, or root resorptions.

#### µCT imaging

The teeth were initially scanned using a µCT system (µCT-50; Scanco Medical, Bassersdorf, Switzerland) with the following parameters: isotropic resolution of 30 μm, scanning vial diameter of 48 mm, voltage of 90 kVp, current of 88 mA, power of 8 W, integration time of 500 ms, and 500 projections per 180°. Scanning was performed perpendicular to the longitudinal axis of the root.

#### CBCT imaging

Subsequently, the teeth were scanned using two protocols with distinct voxel sizes. Scans with a voxel size of 200 μm (acquired using the NewTom 5G system with a FOV of 15 cm × 12 cm; voltage: 110 kVp; current: 3.0 mA) and scans with a voxel size of 80 μm (acquired using the 3D Accuitomo 170 system with a FOV of 4 cm × 4 cm; voltage: 90 kV; current: 5 mA) were obtained.

### Training samples acquisition

To address the significant variation in tooth size and ensure consistent image dimensions for training, the datasets for single-rooted teeth (SR) and molars (M) were preprocessed. CBCT volumes with 200 μm and 80 μm voxel sizes were cropped to dimensions of 64 × 64 and 160 × 160 for SR, and 80 × 80 and 200 × 200 for M, respectively. The depth of the cropped volumes was adjusted according to the tooth length, ensuring complete coverage of the tooth while retaining sufficient background context for segmentation.

In the control group (manual-label-based AI segmentation), two endodontists manually annotated the tooth and root canal structures within the training and validation sets. An experienced endodontist reviewed and refined these annotations using Medical Imaging Interaction Toolkit (MITK, version 2022.04; German Cancer Research Center (DKFZ), Division of Medical Image Computing, Heidelberg, Germany).

In the experimental group (µCT-guided AI segmentation), 3D tooth models with root canal systems were generated from µCT data using a thresholding method in Mimics software (v18.0; Materialise NV, Leuven, Belgium). These tooth masks, containing root canals, were imported into the MITK software and registered to CBCT images using the rigidICP.3D.default algorithm. Following similar registration protocol with previous studies [30], µCT masks were normalized to match the resolution of the CBCT images. Training samples were created by mapping the tooth and root canal masks onto the CBCT images.

For both groups, the registered 3D models derived from µCT data served as the ground truth for the test set. The SR and M samples were divided into a training set (18 teeth), a validation set (5 teeth), and a test set (5 teeth). All data were exported in NIFTI format.

To address the issue that large voxel sizes in the original CBCT images hindered the recognition of fine root canals and compromised segmentation continuity, all images were resampled to smaller voxel dimensions. Specifically, cropped images with an initial voxel size of 200 μm were refined to 100 μm, 80 μm, 50 μm, and 40 μm. Similarly, images starting at 80 μm were interpolated down to 50 μm and 40 μm using linear interpolation. The 3D visualization of RCS was compared and analyzed across different voxel sizes and resampling levels.

### Training and testing of AI segmentation models

**Fig. 1:**
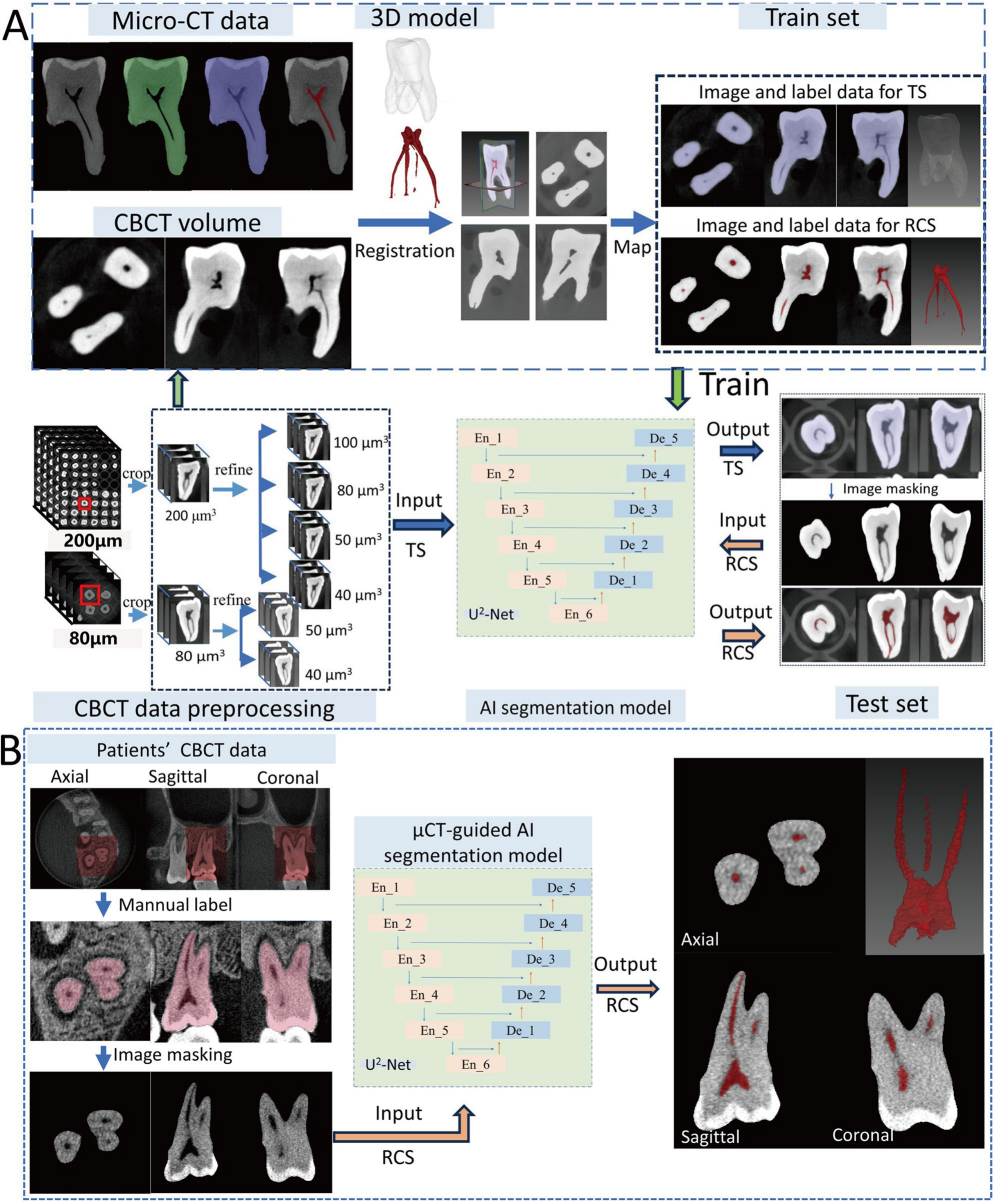
Framework of the proposed two-stage segmentation process. **A** Workflow for Tooth Segmentation (TS) and Root Canal Segmentation (RCS) from Cone-Beam Computed Tomography (CBCT) images of isolated teeth. **B** Workflow for applying the pre-trained model to perform RCS directly on patient CBCT images

The U^2^-Net architecture, a robust deep CNN designed for salient object segmentation, was employed as the AI segmentation model in this study [32]. The model was iteratively trained to optimize performance, with the framework comprising two stages: TS (tooth segmentation) and RCS, as illustrated in Fig. 1A. For the training and validation sets, RCS images were extracted within tooth mask boundaries using MITK software. In the test set, teeth were segmented based on predicted tooth contours.

The DL algorithms and models were implemented using PyCharm Community Edition (Version 2022.2.3 × 64; JetBrains s.r.o., Prague, Czech Republic) and Python (Version 3.9) on a Windows 10 system equipped with NVIDIA RTX A5000 GPU (24GB memory). The PyTorch framework (version 1.7.0; Meta Platforms, Inc., Menlo Park, CA, USA) was utilized for model implementation, with optimization performed using the Adam optimizer at an initial learning rate of 10⁻³. Training was conducted for 400 epochs per group, with durations ranging from 1.3 to 50 h depending on dataset size. The full-sized U^2^-Net architecture was applied, and data augmentation techniques, including random rotation and clipping, were employed to mitigate overfitting.

### Quantitative and qualitative evaluation

The following metrics were used to evaluate the voxel-matching accuracy of the segmentation models:

Dice Similarity Coefficient (DSC):

1 $$\:\begin{array}{c}DSC\:=\:\frac{2\left|{V}_{gt}\cap\:\:{V}_{seg}\right|}{\left|{V}_{gt}\right|+\left|{V}_{seg}\right|}\end{array}$$

Sensitivity (SEN/Recall):

2 $$\:\begin{array}{c}SEN\:=\frac{\left|{V}_{gt}\cap\:{V}_{seg}\right|}{\left|{V}_{gt}\:\right|}\end{array}$$

Intersection over Union (IOU):

3 $$\:\begin{array}{c}IOU=\frac{\left|{V}_{gt}\cap\:{V}_{seg}\right|}{\left|{V}_{gt}\cup\:{V}_{seg}\right|}\end{array}$$

Where $\:{V}_{gt}$ and $\:{V}_{seg}$ represent the voxel sets of the ground truth (label data) and the model segmentation, respectively. Higher values of DSC, SEN, and IOU indicate superior performance. Visual comparisons between manual-label-based and µCT-guided AI segmentation models were conducted using MITK and GOM Inspect Pro software (GOM Software 2022, GOM GmbH, Braunschweig, Germany). Surface deviation analysis was performed for TS (-0.5 mm to + 0.5 mm) and RCS (-0.2 mm to + 0.2 mm).

### RCS from clinical patient CBCT images

In the clinical application of the established mapping relationship between isolated teeth CBCT and µCT, limited FOV CBCT image series were collected from 20 anonymous patients. Scans were acquired using a 3D Accuitomo 170 system (J Morita Mfg. Corp., Kyoto, Japan) with a voxel size of 80 μm³, operating at 90 kV and 5 mA, and a FOV of 4 cm × 4 cm. A total of 29 teeth (17 SR and 12 M) without restorations or fractures were extracted as bounding boxes. Axial image sizes were standardized to 160 × 160 for SR and 200 × 200 for M. Teeth were manually labeled from patient CBCT scans, and regions of interest (ROIs) within tooth contours were extracted for RCS. As depicted in Fig. 1B, the extracted tooth images were input into the pre-trained µCT-guided AI segmentation model for automatic RCS. Results were qualitatively assessed by three endodontists (each with over 8 years of clinical experience) and categorized as “excellent”, “good”, “fair”, or “poor”. This evaluation was based on the anatomical completeness, accuracy, contour continuity and clinical utility of the 3D segmentation models. Excellent: Complete and accurate segmentation of the root canal system, clinically ready for direct use. Good: All major anatomical features identified with only minor inaccuracies, requiring negligible manual refinement. Fair: Basic canal course visible but with notable omissions or errors, necessitating substantial manual correction. Poor: Anatomically incorrect or severely incomplete segmentation, not clinically usable. Disagreements were resolved through consensus discussions.

### Statistical analysis

The normality of the data was assessed using the Shapiro-Wilk test. With normality confirmed, all data are presented as mean ± standard deviation. For paired data following a normal distribution, the paired t-test was applied. A significance level of *p* < 0.05 was adopted for all analyses. All statistical evaluations were performed using SPSS 16.0 software (IBM Corp., Armonk, NY, USA).

## Results

### Performance of the µCT-Guided AI segmentation model across different native voxel sizes

**Fig. 2:**
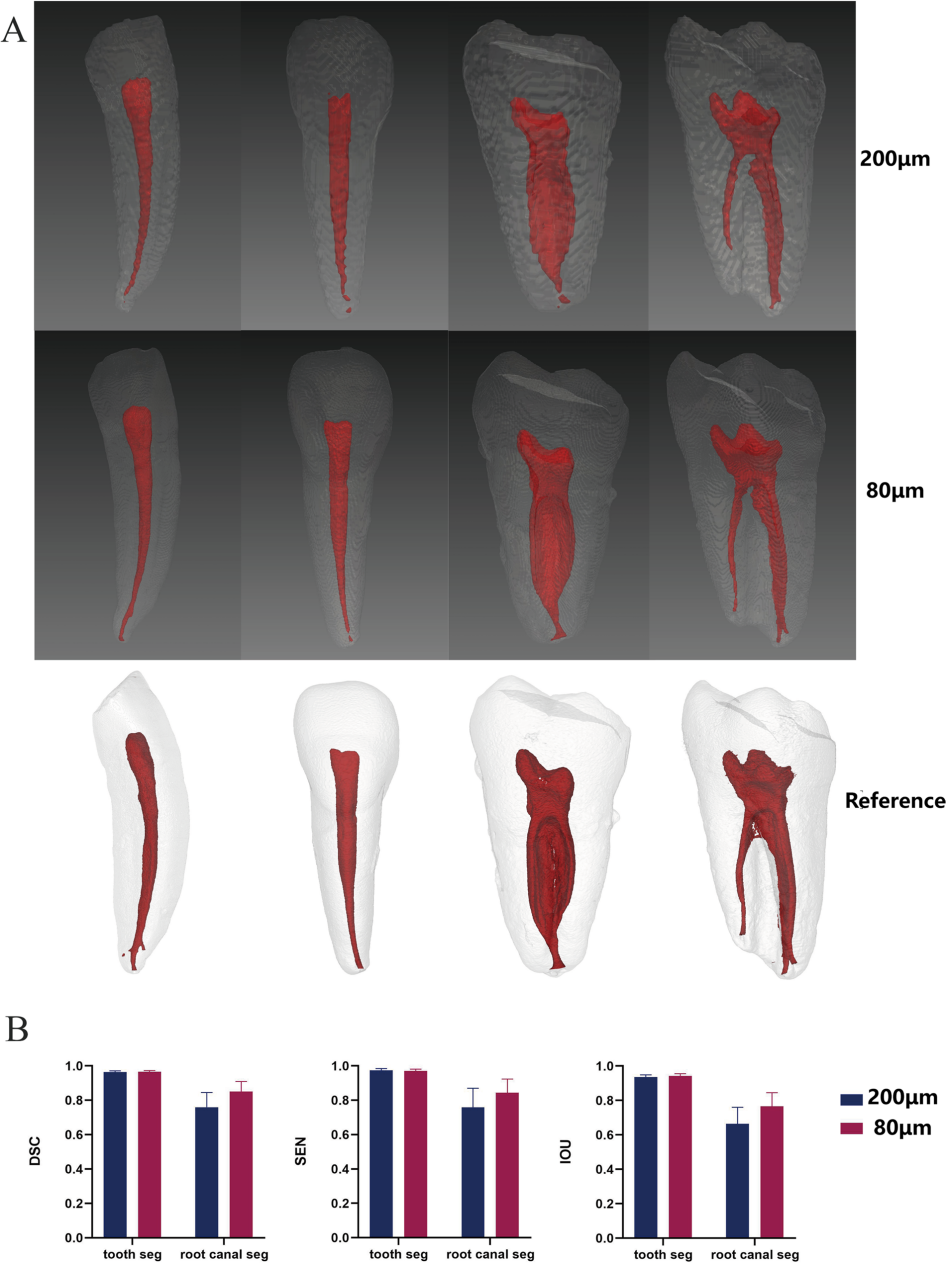
Segmentation outcomes of the μCT-guided AI model applied to the test set across different voxel sizes. **A** Three-dimensional (3D) visualizations of the predicted segmentation morphology alongside the corresponding 3D reference models derived from μCT data. **B** Quantitative analysis of segmentation accuracy, evaluated using Dice Similarity Coefficient (DSC), Sensitivity (SEN), and Intersection over Union (IOU). Data are presented as mean ± standard deviation

Figure 2 illustrates the segmentation results generated by the μCT-guided AI model on native 200 µm and 80 µm CBCT images. The model achieved superior performance in tooth TS across both resolutions. For RCS, the 80 µm group demonstrated better performance in segmenting small root canals near the apex compared to the 200 µm group. No statistical analysis was performed on the differences between segmentation results across different native voxel sizes.

### Comparison of AI segmentation model performances

**Fig. 3:**
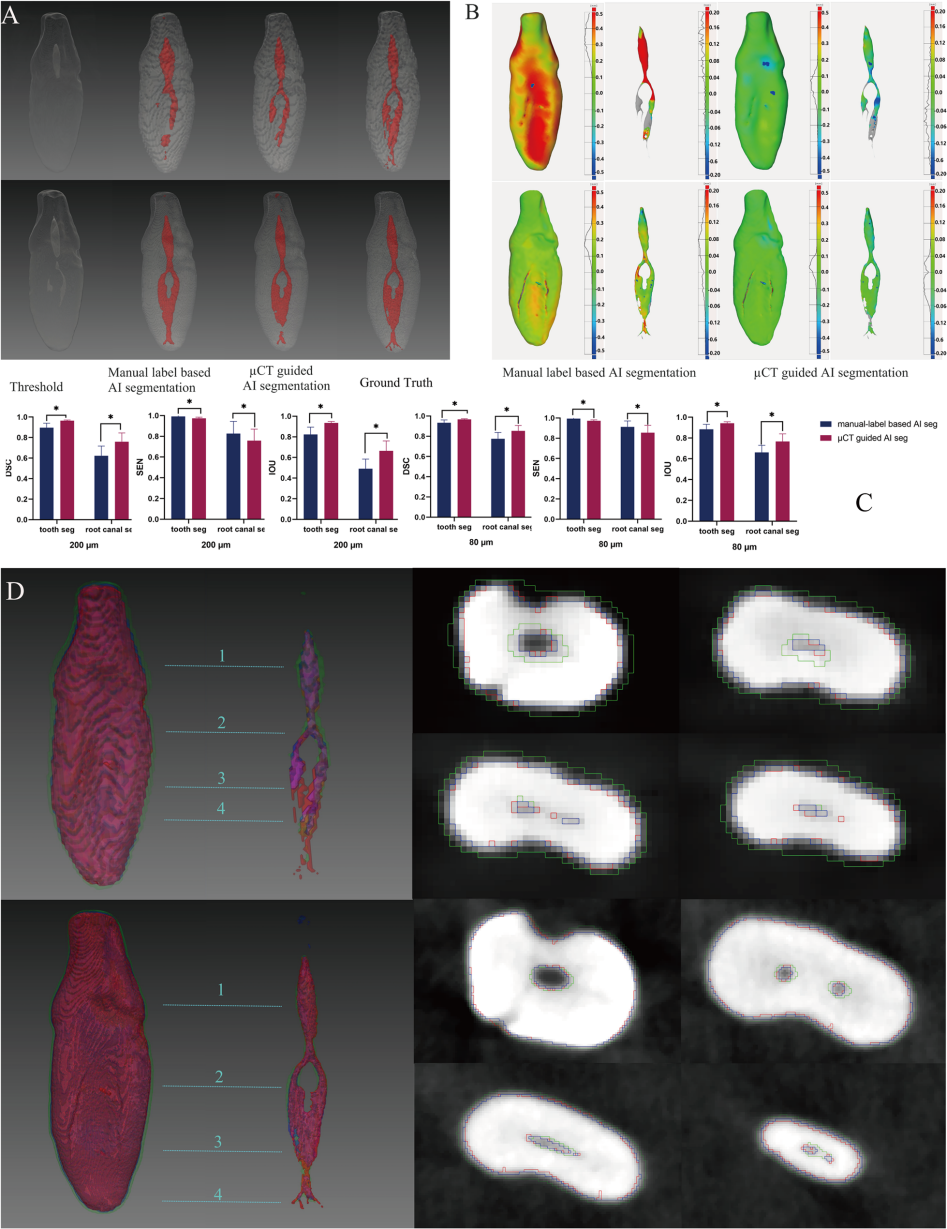
Representative segmentation results analysis conducted on native 200 μm and 80 μm images of a mandibular anterior tooth with a complex root canal using different methods. **A** Comparison between thresholding method and the two types of AI segmentation models. **B** Surface deviation analysis between AI predictions and the ground truth. **C** Quantitative assessment in terms of Dice Similarity Coefficient (DSC), Sensitivity (SEN), and Intersection over Union (IOU), *p< 0.05. (D) 3D superimposition analysis and cross-sectional comparisons at four different slices for the two types of AI segmentation models against the ground truth (red: ground truth, green: manual-label-based AI segmentation, blue: µCT-guided AI segmentation)

Both AI segmentation models consistently outperformed the global thresholding algorithm across different native voxel sizes (Fig. 3). The µCT -guided model achieved significantly higher DSC and IoU values for TS and RCS compared to the manual-label-based model (*p* < 0.05), although it showed a statistically significant decrease in SEN (*p* < 0.05) (Fig. 3C). Variations in root canal diameter and morphology significantly influenced the evaluation metrics, with both models encountering challenges in accurately segmenting small root canals with complex anatomical variations. Specifically, the manual-label-based model tended to overestimate tooth structure boundaries in cases of narrow and irregular root canals, particularly in 200 μm voxel size images, resulting in expanded predicted contours and increased unidentifiable regions (Fig. 3B, D). Surface deviation analysis further revealed that the µCT-guided model demonstrated greater consistency in 80 μm voxel size images, compared to the manual-label-based approach. Deviations were predominantly observed in regions with fine root canals and accessory canals (Fig. 3B).

### Effect of voxel-size resampling on segmentation results

**Fig. 4:**
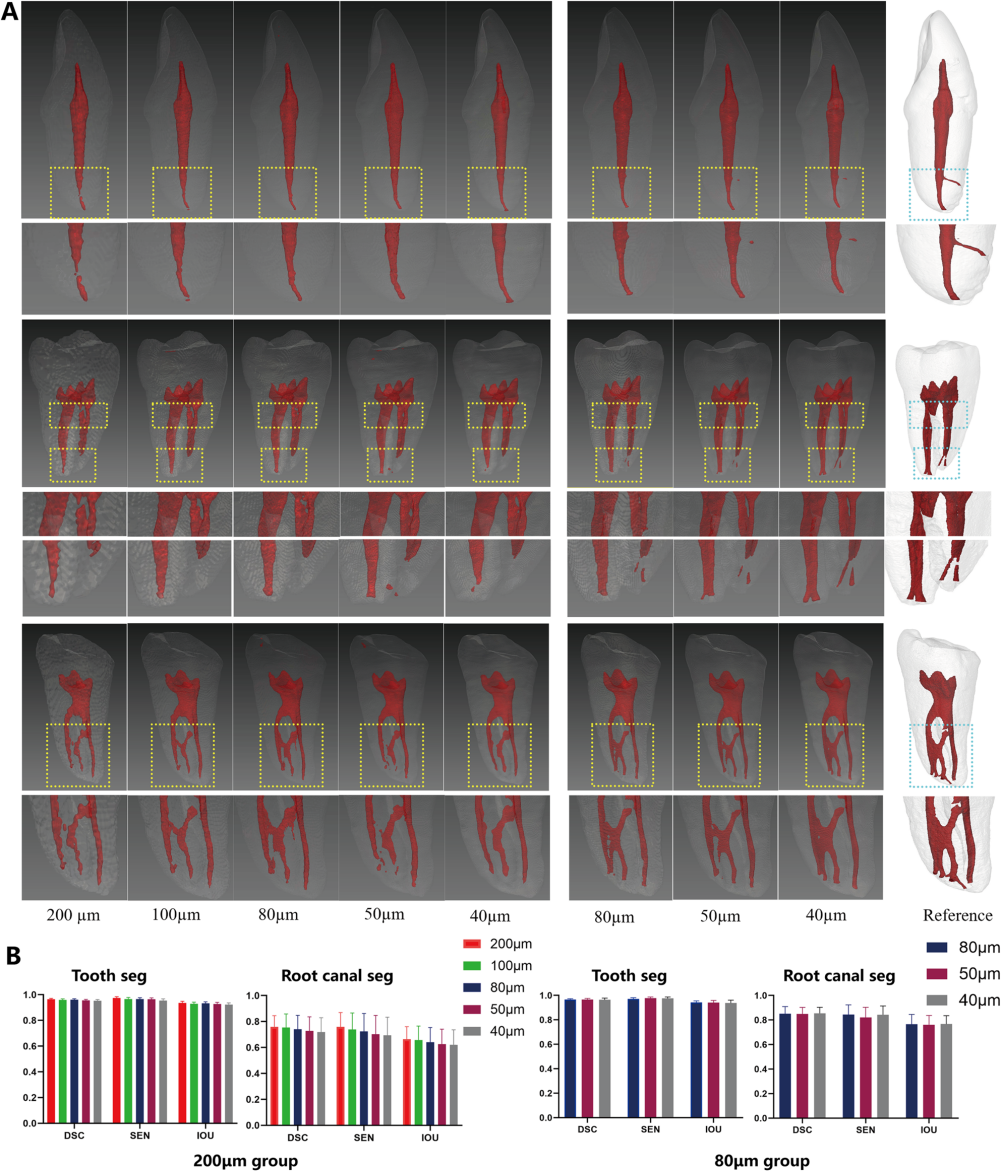
Effect of voxel-size resampling on segmentation performance across images with different native voxel sizes (200 μm vs. 80 μm). **A** 3D visualization of µCT-guided AI segmentation results at progressively voxel-size resampling. The reference column shows the corresponding µCT-based 3D model. **B** Quantitative analysis of segmentation metrics of Dice Similarity Coefficient (DSC), Sensitivity (SEN), and Intersection over Union (IOU) across interpolation levels for both 200 μm and 80 μm groups

Figure 4A presents 3D visualizations of voxel-size resampling using the µCT-guided AI model. In the 200 μm group, a single resampling step to 100 μm enabled the detection of a discontinuous apical canal that was invisible at the native resolution. However, further resampling to 40 μm only smoothed boundaries without recovering additional fine structures like accessory canals. Conversely, in the 80 μm group, resampling to 40 μm enhanced the segmentation of accessory canals and isthmuses. At matched voxel sizes, the 80 μm group consistently yielded superior RCS quality than 200 μm group.

Quantitative analysis in Fig. 4B reveals that TS performance metrics (DSC ≥ 0.9488, SEN ≥ 0.9533, IOU ≥ 0.9181) remained consistently high across all conditions. However, in 200 μm group, resampling to smaller voxel sizes led to a decrease in all quantitative evaluation metrics for RCS. In the 80 μm group, the quantitative evaluation metrics for RCS remained stable, showing no marked declining trend across the tested range.

### RCS from patient CBCT images

**Fig. 5:**
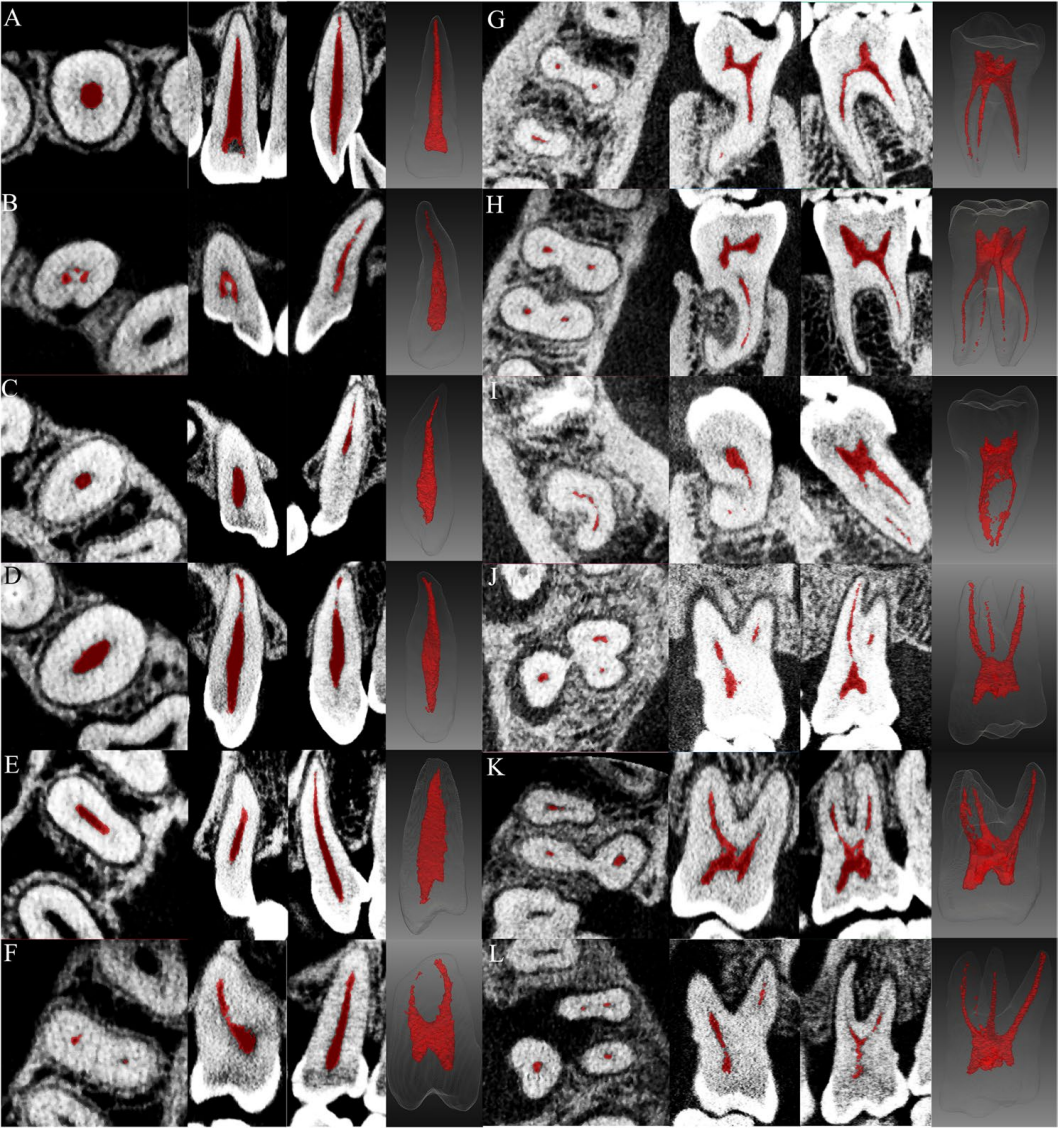
Predicted root canals from patient’s CBCT images: root canal segmentation results displayed in coronal, sagittal, and axial planes, alongside their reconstructed 3D models. **A**-**F** Single-rooted teeth (SR). **G**-**L** Molars (M)

In SR, 53% (9/17) of cases achieved an “excellent” rating, while 41% (7/17) were classified as “good,” with no instances falling under the category of “poor.” The segmentation of root canals up to the apical foramen was successfully achieved in common root canal types, as shown in Fig. 5A-F. Even challenging cases, such as C-shaped canals (Fig. 5B) and accessory canals (Fig. 5D), were accurately identified. False-positive regions occasionally appeared during the root canal prediction process in M, mainly in the coronal dentin under the enamel. The ImageJ/Fiji software (National Institutes of Health, Bethesda, MD, USA) was utilized along with the Find Connected Regions plugin to separate predicted voxels into individual objects, enabling the removal of false-positive objects and the extraction of root canals. As illustrated in Fig. 5G-L, only a few extremely tiny root canals failed to be continuously segmented; these segments were difficult to discern even with the naked eye. After post-processing, the evaluation results indicated that 66.7% (8/12) of cases achieved an “excellent” rating, while the remaining 33.3% (4/12) were rated as “good”.

## Discussion

The µCT-guided model achieved superior DSC and IoU but lower SEN than the manual-label-based model. The observed divergence reflected the distinct focus of each metric. SEN quantifies the model’s ability to avoid under-segmentation (i.e., capturing true canal voxels), whereas DSC and IoU are composite measures that balance sensitivity with precision (i.e., avoiding false positives). This discrepancy stemd fundamentally from the different training references. The manual-label-based model, trained on CBCT-derived annotations limited by image contrast, adopted a conservative segmentation strategy. It tended to outline broader boundaries for fine or irregular canals to minimize misses, thereby increasing SEN at the expense of precision, which in turn lowered DSC and IoU (Fig. 3B, D). In contrast, the µCT-guided model was trained on high-resolution masks obtained via µCT thresholding, which provide an anatomically precise standard. Consequently, this model prioritized precision and morphological fidelity, resulting in higher DSC and IoU, even if it occasionally omitted the faintest voxels identified by the manual approach. Thus, the µCT-guided framework ensures objectively accurate segmentation against a high-resolution standard.

**Fig. 6:**
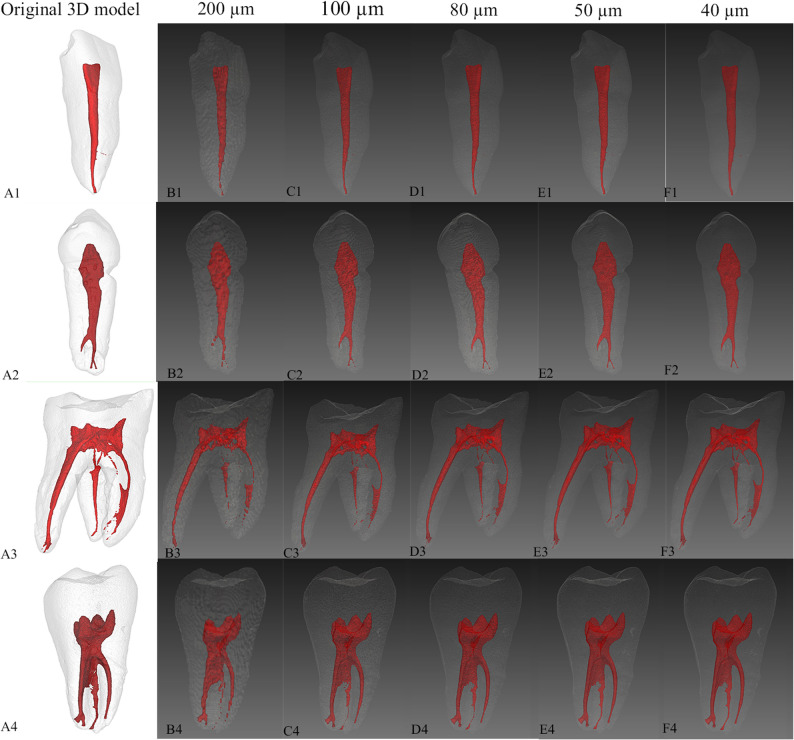
Variation of ground truth 3D models following registration to CBCT images with different voxel sizes. In four representative teeth, registered models retain more detail at finer voxel sizes; larger voxels cause morphological simplification. (A1–A4) High-resolution reference models reconstructed directly from µCT data after thresholding. (B1–B4) The same µCT-derived masks after registration to simulated CBCT images with a 200 μm voxel size. (C1–C4) Registered models corresponding to a 100 μm voxel size. (D1–D4) Registered models corresponding to an 80 μm voxel size. (E1–E4) Registered models corresponding to a 50 μm voxel size. F1–F4: Registered models corresponding to a 40 μm voxel size

Resampling mitigates issues from large voxel sizes but cannot add new information. Excessive interpolation introduces artifactual smoothing which was confirmed in the 200 μm group, where resampling to 40 μm merely smoothed boundaries, failing to recover additional fine structures such as accessory canals. Additionally, in 200 μm group, resampling to smaller voxel sizes led to a decrease in all quantitative evaluation metrics for RCS, which appeared to contradict the positive qualitative results shown in Fig. 4A. This seemingly contradictory outcome could be explained by the process of registering high-resolution tooth or root canal masks (generated from µCT via thresholding) to CBCT images with different voxel sizes. When high-resolution masks are registered to a lower-resolution CBCT image, the resulting ground truth is resampled to match the voxel size of the target image. Consequently, finer anatomical details may be lost when registering to larger-voxel CBCT images, whereas more subtle morphological features can be preserved when aligning with smaller-voxel CBCT images (Fig. 6). Consequently, the ground truth itself varies across voxel-size groups, which explains why quantitative metrics and qualitative observations may not align directly. In the case of 80 μm group, which already possess a relatively small native voxel size, delicate structures such as fine root canals are largely retained. As a result, the differences between ground truth masks across the tested resample range are minimal, which accounts for the lack of decline change in RCS evaluation metrics observed in this experiment. Due to these inherent variations in the ground truth following registration across different voxel sizes, no statistical comparison was performed between the segmentation results from different voxel-size groups.

The accuracy of this framework is influenced not only by the µCT standard but also by the resolution of the target CBCT images. The µCT-derived masks were down-sampled during registration to match various CBCT voxel sizes. Our experiments indicate that while judicious image interpolation can help recover fine structures obscured by large voxels, excessive resampling may overly smooth features and degrade quantitative accuracy [33]. This underscores that the effective resolution of the ground truth is pivotal. Our findings suggest that for root canal segmentation, a voxel size of 80 μm³ strikes a practical balance, adequately representing most anatomical details without prohibitive computational cost. Notably, RCS performance proved more sensitive to native scan resolution than TS, justifying the selection of higher-resolution (80 μm) protocols for subsequent clinical application.

**Fig. 7:**
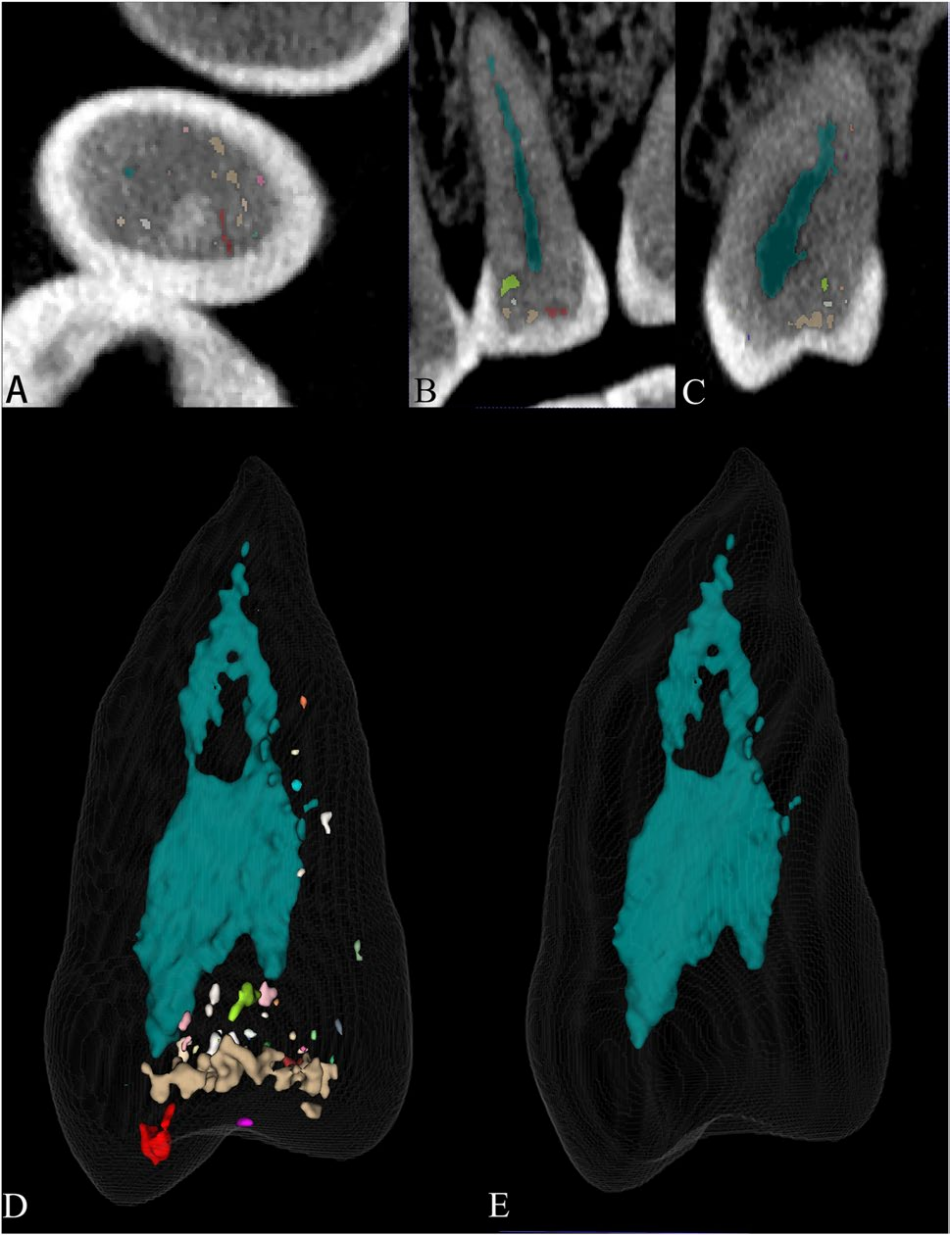
Illustration of false-positive over-segmentation and its correction in a premolar root canal segmentation. **A** Axial, (**B**) Coronal, and (**C**) Sagittal CBCT views with the initial AI-based segmentation overlaid. The root canal system is rendered in blue, while other colors indicate a representative region of false-positive over-segmentation. **D** 3D rendering of the initially segmented canal system. **E** The corrected 3D model following automated post-processing for the removal of false-positive voxels using ImageJ

Several limitations of this study point to valuable directions for future work. First, our dataset, though containing representative complex anatomy such as C-shaped canals, is limited in size (*n* = 56) and scope. Features like root curvature were not graded, which may constrain model generalizability. Future efforts should expand to larger, multicentric datasets with detailed anatomical annotations to improve robustness. Moreover, there should be an increased emphasis on automating tooth instance segmentation using DL to alleviate the tedious process of manually delineating tooth boundaries in clinical CBCT images. Secondly, the use of patient CBCT data for external validation of the µCT-guided CBCT segmentation model introduced heterogeneity compared to the isolated teeth used in the training data. This heterogeneity was particularly evident during the segmentation of multi-rooted premolars and molars, where occasional false-positive regions emerged and hindered automated RCS (Fig. 7). However, such heterogeneity was rarely observed in the single-rooted anterior teeth included in this study. The discrepancy in RCS performance may be attributed to the dense bone tissue surrounding molars in CBCT images, as well as the relatively narrower diameter of their root canals compared to single-rooted anterior teeth, resulting in reduced contrast between dentin and root canals. Consequently, the limitations of global threshold segmentation methods became more pronounced. Further research is necessary to develop more advanced methods to replace the global thresholding approach currently used in constructing 3D tooth models from µCT data. Finally, in this study, we employed the same registration protocol similar with previous studies [30], a quantitative assessment of the registration error specific to our dataset was not performed. Future work would benefit from incorporating such metrics (e.g., target registration error using fiducial markers) to provide dataset-specific validation and further strengthen the methodological rigor.

## Conclusions

This study validated a clinically viable solution for root canal segmentation by developing a µCT-guided AI model that automated the process on CBCT scans. The model’s superiority over traditional global thresholding techniques and manual label-based approaches confirmed the value of high-fidelity ground truth for training. Our findings demonstrated the advantages of optimal image resolution and careful resampling in capturing intricate canal morphology. The framework’s effective performance on patient scans confirmed its potential to directly translate into clinical workflows, simplifying treatment planning and contributing to more predictable outcomes in endodontic therapy.

## Data Availability

Most of data generated or analyzed during this study are included in this article. The original datasets used and/or analyzed during the current study are available from the corresponding author on reasonable request.

## Abbreviations

CBCT: Cone-beam computed tomography
µCT: Micro-computed tomography
AI: Artificial intelligence
FOV: Field of view
SR: Single rooted teeth
M: Molars
3D: Three-dimensional
DL: Deep Learning
CNN: Convolutional Neural Network
DSC: Dice Similarity Coefficient
SEN: Sensitivity
IOU: Intersection over Union
TS: Tooth segmentation
RCS: Root canal segmentation
